# Prediction and diagnosis of interval metastasis after neoadjuvant chemoradiotherapy for oesophageal cancer using ^18^F-FDG PET/CT

**DOI:** 10.1007/s00259-018-4011-6

**Published:** 2018-04-16

**Authors:** Lucas Goense, Jelle P. Ruurda, Brett W. Carter, Penny Fang, Linus Ho, Gert J. Meijer, Richard van Hillegersberg, Wayne L. Hofstetter, Steven H. Lin

**Affiliations:** 1 Department of Radiation Oncology, The University of Texas MD Anderson Cancer Center, 1515 Holcombe Blvd, Houston, TX 77030 USA; 2 Department of Radiation Oncology, University Medical Center Utrecht, Utrecht University, Heidelberglaan 100, 3584 CX Utrecht, The Netherlands; 3 Department of Surgery, University Medical Center Utrecht, Utrecht, The Netherlands; 4 Department of Diagnostic Radiology, The University of Texas MD Anderson Cancer Center, 1515 Holcombe Blvd, Houston, TX 77030 USA; 5 Department of Gastrointestinal Medical Oncology, The University of Texas MD Anderson Cancer Center, 1515 Holcombe Blvd, Houston, TX 77030 USA; 6 Department of Thoracic and Cardiovascular Surgery, The University of Texas MD Anderson Cancer Center, 1515 Holcombe Blvd, Houston, TX 77030 USA

**Keywords:** Oesophageal cancer, ^18^F-FDG PET/CT, Cancer staging, Chemoradiotherapy, Esophagectomy

## Abstract

**Objective:**

During neoadjuvant chemoradiotherapy for oesophageal cancer, or in the interval prior to surgery, some patients develop systemic metastasis. This study aimed to evaluate the diagnostic performance of ^18^F-FDG PET/CT for the detection of interval metastasis and to identify predictors of interval metastases in a large cohort of oesophageal cancer patients.

**Methods:**

In total, 783 consecutive patients with potentially resectable oesophageal cancer who underwent chemoradiotherapy and pre- and post-treatment ^18^F-FDG PET/CT between 2006 and 2015 were analyzed from a prospectively maintained database. Diagnostic accuracy measures were calculated on a per-patient basis using histological verification or clinical follow-up as a reference standard. Multivariable logistic regression analysis was performed to determine pre-treatment predictors of interval metastasis. A prediction score was developed to predict the probability of interval metastasis.

**Results:**

Of 783 patients that underwent ^18^F-FDG PET/CT restaging, 65 (8.3%) were found to have interval metastasis and 44 (5.6%) were deemed to have false positive lesions. The resulting sensitivity and specificity was 74.7% (95% CI: 64.3–83.4%) and 93.7% (95% CI: 91.6–95.4%), respectively. Multivariable analysis revealed that tumor length, cN status, squamous cell tumor histology, and baseline SUV_max_ were associated with interval metastasis. Based on these criteria, a prediction score was developed with an optimism adjusted C-index of 0.67 that demonstrated accurate calibration.

**Conclusions:**

^18^F-FDG PET/CT restaging detects distant interval metastases in 8.3% of patients after chemoradiotherapy for oesophageal cancer. The provided prediction score may stratify risk of developing interval metastasis, and could be used to prioritize additional restaging modalities for patients most likely to benefit.

## Introduction

Oesophageal cancer affects more than 450,000 people annually, and is the sixth leading cause of cancer-related mortality worldwide [1]. Currently, surgical resection of the esophagus preceded by neoadjuvant chemoradiotherapy is the standard of care for patients with non-metastasized oesophageal cancer [1–3]. Definitive chemoradiotherapy is the preferred approach for inoperable locally advanced oesophageal cancer [4, 5]. In consequence of the duration of chemoradiotherapy and subsequent waiting time to surgery, systemic interval metastases may develop that were not visible during baseline staging [6–8]. In these patients curative treatment is no longer possible [9].

Currently, there is disagreement between guidelines as to whether all patients should be restaged after chemoradiotherapy [10–12]. In different international guidelines, routine restaging with computed tomography and integrated ^18^F-fluorodeoxyglucose positron emission tomography (^18^F-FDG PET/CT) is not recommended [11], or partially recommended for patients with cT3–4 or cN1–3 tumors [12]. To the contrary, the National Comprehensive Cancer Network advises restaging for all patients who receive preoperative chemoradiotherapy [10]. At present, little is known about which patients are at risk of developing interval metastases.

Several small studies have assessed the role of ^18^F-FDG PET/CT for pre-surgical restaging after neoadjuvant therapy, with reported incidence rates of interval metastases varying from 2% up to 26% [13, 14]. However, most studies did not report diagnostic accuracy measures (i.e. sensitivity, specificity) and included only a small number of patients [6–8]. Also, studies that have assessed clinical predictors for interval metastases are scarce [15]. Accurate prediction of disease progression during and shortly after chemoradiotherapy would enable surveillance tailored to each patient’s underlying risk of developing systemic disease.

The aim of the current study was two-fold. First, to quantify the incidence of interval metastases after chemoradiotherapy and evaluate the diagnostic performance of ^18^F-FDG PET/CT for the detection of interval metastases in a large cohort of patients. Second, to identify pre-treatment clinical predictors for interval metastases.

## Methods

This retrospective study was approved by the institutional review board of the MD Anderson Cancer Center and the requirement to obtain informed consent was waived. The study was conducted in accordance with the Health Insurance Portability and Accountability Act (HIPAA), the checklist from the Transparent Reporting of a multivariable prediction model for Individual Prognosis or Diagnosis statement (http://www.tripod-statement.org) [16], and the checklist from the STAndards for the Reporting of Diagnostic accuracy studies (STARD) statement (http://www.stard-statement.org) [17].

### Study population

**Fig. 1:**
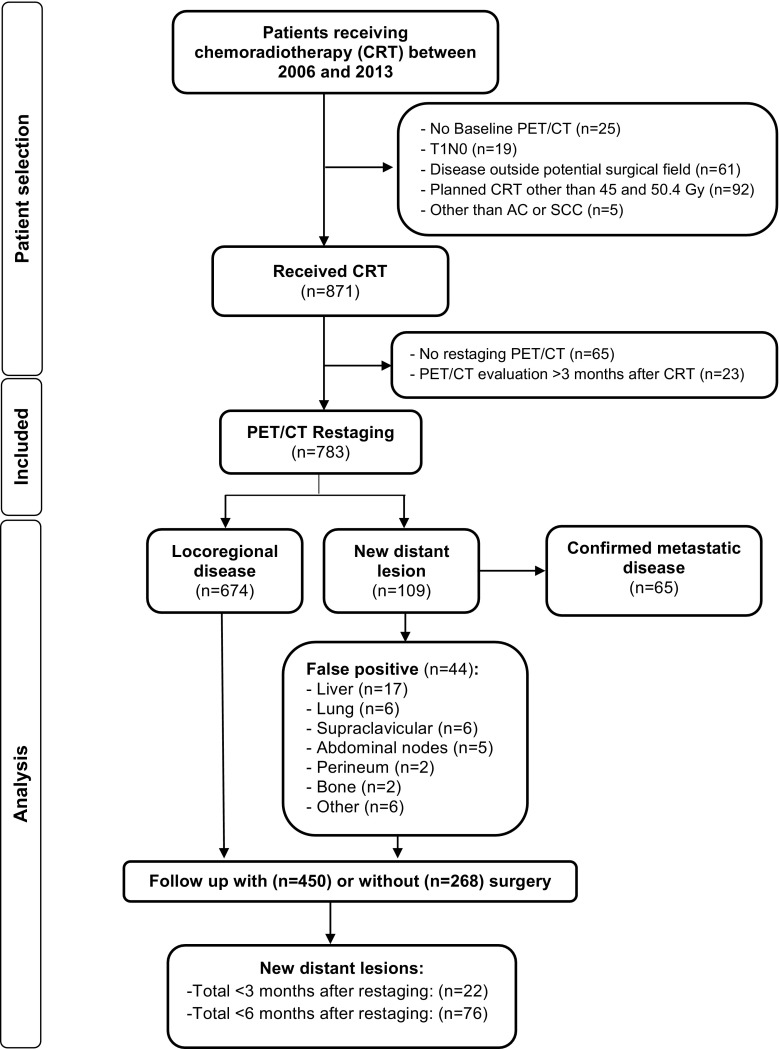
Flowchart of the study

Data from consecutive patients with biopsy-proven adenocarcinoma or squamous cell carcinoma of the esophagus who received chemoradiotherapy (with or without surgery) at the University of Texas MD Anderson Cancer Center from January 2006 to July 2013 were extracted from a prospective collected departmental registry. Inclusion criteria were; non-metastatic potentially resectable oesophageal cancer (cT1N + M0 or cT2-4aM0 with nodes in the anatomic region of a 2-field lymph node dissection), scheduled radiation dose of 45 or 50.4 Gy with concurrent chemotherapy, staging with ^18^F-FDG PET/CT before and after chemoradiotherapy. Patients were excluded if the time interval between completion of chemoradiotherapy and ^18^F-FDG PET/CT restaging was more than 3 months. The flow of patient selection is summarized in Fig. 1. Disease was staged in accordance with the 7th edition of the International Union Against Cancer for cTNM-classification [18]. Initial diagnostic work-up included endoscopy with biopsy, endoscopic ultrasound (including fine-needle aspiration if indicated), and ^18^F-FDG PET/CT.

### Treatment protocol

Chemoradiotherapy treatment generally consisted of a fluoropyrimidine (IV or oral) with either platinum- or taxane-based chemotherapy with concurrent radiotherapy (45 or 50.4 Gy in fractions of 1.8 Gy) (Table 1). Four to 6 weeks after completion of chemoradiotherapy, all patients underwent re-staging procedures and were discussed in multidisciplinary tumor conferences. Patients that were deemed eligible for surgical treatment proceeded to oesophagectomy. Surgical treatment consisted of transthoracic oesophagectomy combined with lymphadenectomy.

**Table 1:** Patient and treatment-related characteristics and their association with interval metastasis detected by ^18^F-FDG PET/CT after neoadjuvant chemoradiotherapy

Characteristic		All Patients (*n* = 783)		Potentially resectable disease (*n* = 718)		Systemic interval metastases (*n* = 65)		*p* value
		n	%	n	%	n	%	
Gender	Male	675	86.2%	619	91.7%	56	8.3%	0.990
	Female	108	13.8%	99	91.7%	9	8.3%	
Age at diagnosis	<65 Years	425	54.3%	386	90.8%	39	9.2%	0.334
	≥65 years	358	45.7%	332	92.7%	26	7.3%	
Body mass index	<25 kg/m^2^	390	49.8%	351	90.0%	39	10.0%	0.086
	≥25 kg/m^2^	393	50.2%	367	93.4%	26	6.6%	
ECOG performance status	0	283	36.1%	262	92.6%	21	7.4%	0.501
	1–2	500	63.9%	456	91.2%	44	8.8%	
Weight loss	<10%	615	78.5%	570	92.7%	45	7.3%	0.056
	≥10%	168	21.5%	148	88.1%	20	11.9%	
Histology	AC	672	85.8%	621	92.4%	51	7.6%	0.076
	SCC	111	14.2%	97	87.4%	14	12.6%	
Histologic differentiation grade^a^	Good/Moderate	363	46.4%	339	93.4%	24	6.6%	0.111
	Poor	420	53.6%	379	90.2%	41	9.8%	
Signet ring cell adenocarcinoma	No	671	85.7%	617	92.0%	54	8.0%	0.529
	Yes	112	14.3%	101	90.2%	11	9.8%	
EUS-based tumor length	<4.0 cm	210	26.8%	204	97.1%	6	2.9%	0.001
	≥4.0 cm	573	73.2%	514	89.7%	59	10.3%	
Nontraversability by EUS	No	645	82.4%	595	92.2%	50	7.8%	0.228
	Yes	138	17.6%	123	89.1%	15	10.9%	
Tumor Location	Upper or middle	103	13.2%	93	90.3%	10	9.7%	0.309
	Distal or GEJ	680	86.8%	625	91.9%	55	8.1%	
SUV_max_ primary tumor at baseline	<9.6	410	60.0%	389	94.9%	21	5.1%	0.001
	≥9.6	373	40.0%	329	88.2%	44	11.8%	
Clinical T status (seventh)^b^	IB/II	90	11.5%	86	95.6%	4	4.4%	0.159
	III/IVa	693	88.5%	632	91.2%	61	8.8%	
Clinical N status (seventh)^b^	cN0	268	34.2%	260	97.0%	8	3.0%	<0.001
	cN+	515	65.8%	458	88.9%	57	11.1%	
Maximum Lymph node diameter^c^	<1.0 cm	542	69.2%	507	93.5%	35	6.5%	0.005
	≥1.0 cm	241	30.8%	211	87.6%	30	12.4%	
PET avid nodes at baseline	*m*N0	480	61.3%	448	93.3%	32	6.7%	0.037
	*m*N1	303	38.7%	270	89.1%	33	10.9%	
Total radiation dose (Gy)	45.0	49	6.3%	43	87.8%	6	12.2%	0.301
	50.4	734	93.7%	675	92.0%	59	8.0%	
Radiation treatment modality	3-D CRT	6	0.8%	5	83.3%	1	16.7%	0.492
	IMRT	505	64.5%	460	91.1%	45	8.9%	
	Proton Therapy	272	34.7%	253	93.0%	19	7.0%	
Chemotherapy regimen	Oxaliplatin / 5-FU	236	30.1%	223	94.5%	13	5.5%	0.286
	Docetaxel / 5-FU	265	33.8%	238	89.8%	27	10.2%	
	Capecitabine / 5-FU	167	21.3%	152	91.0%	15	9.0%	
	Other	115	14.7%	105	91.3%	10	8.7%	

^a:^Determined in pre-treatment biopsy ^b:^Classified according to the 7th edition of the International Union Against Cancer (UICC) tumor-node-metastasis (TNM) classification [18]; ^c:^Lymph node diameter was measured in the short axis by an experienced radiologists on the axial CT images; ECOG: Eastern Cooperative Oncology Group; AC: adenocarcinoma; SCC: squamous cell carcinoma; EUS: endoscopic ultrasonography; SUV: standardized uptake value

### Image acquisition and analysis

Patients were scanned before and after completion of chemoradiotherapy on a dedicated PET/CT system (Discovery RX, ST, or STE; GE Medical Systems, Milwaukee [WI], USA). After fasting for at least 6 h, patients were injected with ^18^F-FDG (555–740 MBq). An unenhanced CT was acquired for attenuation correction purposes (120 kV peaks, 300 mA, 0.5 s rotation, pitch of 1.375, slice thickness 3.75 mm, and slice interval 3.27 mm). PET scans were acquired 60–90 min after administration of ^18^F-FDG in either two-dimensional (2D) or three-dimensional (3D) acquisition mode.

PET/CT interpretations rendered as part of the clinical care were extracted from the original PET/CT reports. All ^18^F-FDG PET/CT images were reviewed by experienced nuclear medicine radiologists who were aware of patients’ information and previous clinical findings. The images were evaluated for the presence of new lesions with non-physiological ^18^F-FDG accumulation. Suspicious lesions on CT scans with increased focal ^18^F-FDG uptake were indicated as malignant. ^18^F-FDG PET/CT images were interpreted as positive for interval metastasis when new malignant lesions were found outside the anatomic dissection plane of an oesophagectomy combined with a two-field lymphadenectomy.

### Reference standard

A composite reference standard combining histologic proof and/or imaging follow-up was used to confirm the disease status of patients after restaging with ^18^F-FDG PET/CT. Histologically verified PET/CT-positive lesions or lesions showing an increase in size or ^18^F-FDG uptake on subsequent radiological follow-up were considered as true-positive (TP). Clinical follow-up was used as reference standard for patients with a negative ^18^F-FDG PET/CT during restaging. Patients were followed every 3 months during the first year after treatment, which included physical examination, blood tests, and ^18^F-FDG PET/CT scans. The restaging ^18^F-FDG PET/CT was considered false negative (FN) in case patients developed new metastatic disease within 3 months after the initial restaging ^18^F-FDG PET/CT scan. Patients without confirmed systemic disease progression during follow-up were considered as true-negative (TN).

### Pre-treatment predictors

All patient, tumor, and treatment-related characteristics as reported in Table 1 were derived from the prospective collected departmental registry. Initial selection of predictors for interval metastasis detected by ^18^F-FDG PET/CT restaging were pre-specified based on previous literature to prevent overfitting of the model. Categories were based on previously published cut-off points or estimated by receiver operating characteristic (ROC) curve analysis while maximizing sensitivity and specificity. Clinical factors available before initiation of treatment that have previously been identified as prognostic factors in oesophageal cancer included gender [19], age (dichotomized into <65 and ≥ 65) [20], Histology (adenocarcinoma versus squamous cell carcinoma [3, 20], histologic differentiation grade (good/moderate versus poor) [20, 21], signet ring cell adenocarcinoma [22, 23], EUS-based tumor length (dichotomized into <4.0 cm and ≥ 4.0 cm) [24, 25], nontraversability by EUS [15, 24], tumor location (upper/middle versus distal or gastro-oesophageal junction) [18], clinical T-status (T1b-2 versus T3–4) [19, 20], clinical N status (N0 versus N1–3) [20, 21], maximum lymph node diameter measured on axial CT image (<1.0 cm versus ≥1 cm) [26, 27], and ^18^F-FDG avid nodes at baseline PET [15]. The maximum standardized uptake value (SUV_max_) of the primary tumor was dichotomized into <9.6 and ≥ 9.6 based on ROC curve analysis.

### Statistics

Patient and treatment-related characteristics were described as frequencies with percentages for categorical variables, mean with standard deviation (SD) for normally distributed variables and median with range for skewed distributions. Sensitivity, specificity, positive predictive value (PPV), negative predictive value (NPV) and accuracy of ^18^F-FDG PET/CT for the detection of interval metastasis were calculated with 95% confidence interval (CI) on a per-patient basis. Kaplan-Meier curves were used to assess overall survival, and survival differences were evaluated using the log-rank test. Statistical analysis was performed using SPSS version 24.0 (IBM Corp., Armonk, NY) and R 3.1.2 open-source software (http://www.R-project.org, ‘rms’ package). A *p*-value of <0.05 was considered statistically significant.

#### Model development

The association between clinical characteristics and interval metastasis was studied using the chi-square test. All potential prespecified predictors for interval metastasis were included in a multivariable logistic regression model. The initial logistic regression model was reduced using backward stepwise elimination based on Akaike Information Criteria. The discriminative ability of the final model was evaluated using receiver operating characteristics curve analysis providing the concordance statistic (C-statistic). For internal validation the model was subjected to 200 bootstrap resamples to calculate the optimism of the model and the shrinkage factor, after which the C-statistic and the β-coefficients were adjusted. A practical scoring system was developed using the *beta*-regression coefficients of the predictors that remained in the final model. Calibration of the model was evaluated by plotting the mean predicted probability of interval metastasis versus the observed percentage of interval metastasis for each level of the prediction score.

## Results

In the study period, a total of 783 patients diagnosed with oesophageal cancer who met our inclusion and exclusion criteria underwent chemoradiotherapy followed by a restaging ^18^F-FDG PET/CT scan (Fig. 1). The distribution of patient, tumor, and treatment-related characteristics are summarized in Table 1. The study population had a mean age of 62.5 years (SD: 10.6 years), and the majority of patients were male (86.2%). The predominant histologic tumor type was adenocarcinoma (85.8%), and the most common clinical tumor stage was cT3 (87.1%). The mean time interval between completion of chemoradiotherapy and ^18^F-FDG PET/CT restaging was 41.3 days (SD: 10.7). After completion of chemoradiotherapy, 450 (57.5%) patients underwent oesophageal resection.

### Diagnostic accuracy

**Fig. 2:**
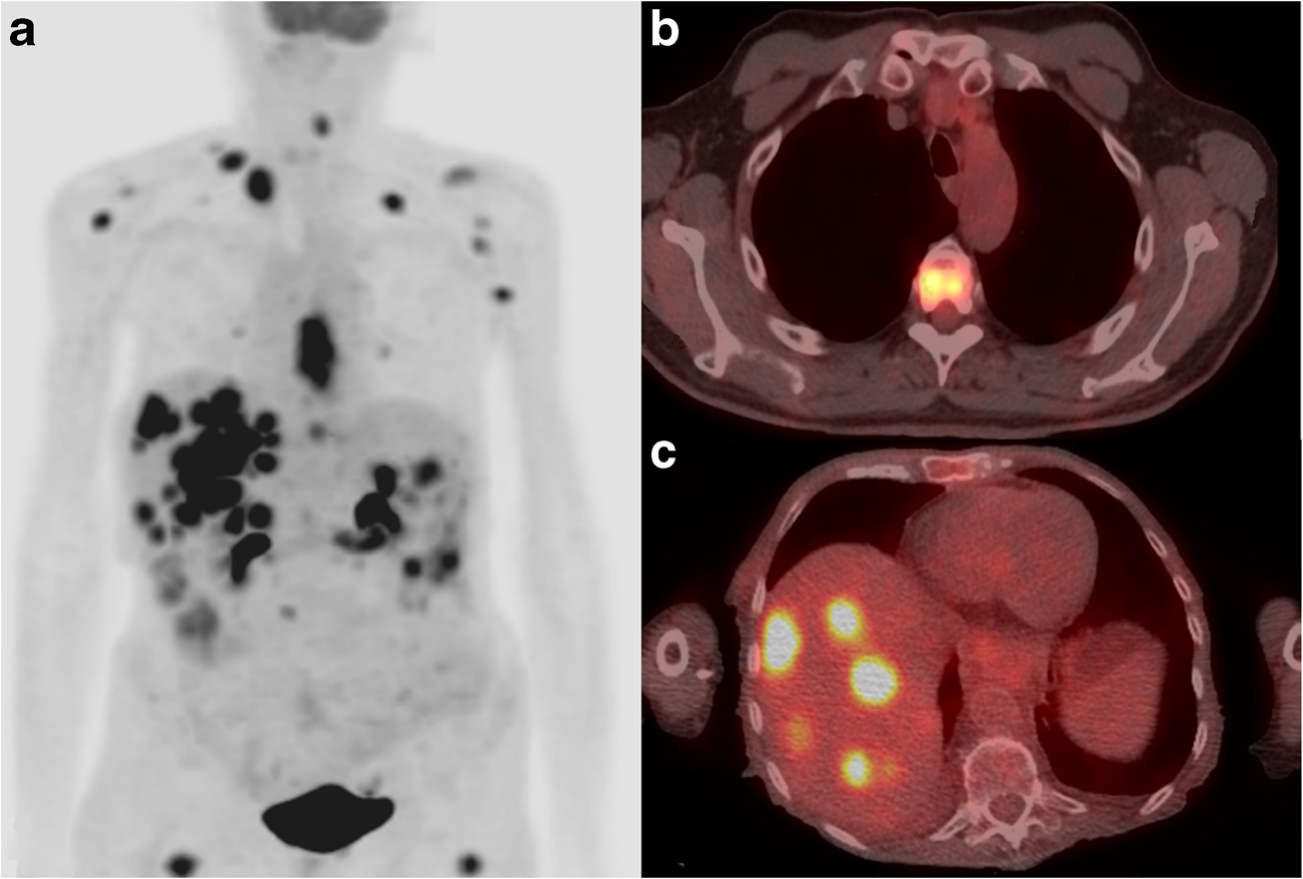
Examples of true positive metastatic lesions detected by ^18^F-FDG PET/CT restaging. (a/c): 80-year-old woman with adenocarcinoma of the distal esophagus treated with chemoradiation. The maximum intensity projection PET image shows multiple hypermetabolic foci of the liver and multiple soft tissue lesions that were confirmed malignant with follow-up scans. (b): 65-year-old male with squamous cell carcinoma of the distal esophagus who had undergone chemoradiotherapy. The PET/CT image showed ^18^F-FDG accumulation in the liver and in the thoracic spine at T5. Follow-up CT showed disease progression

**Fig. 3:**
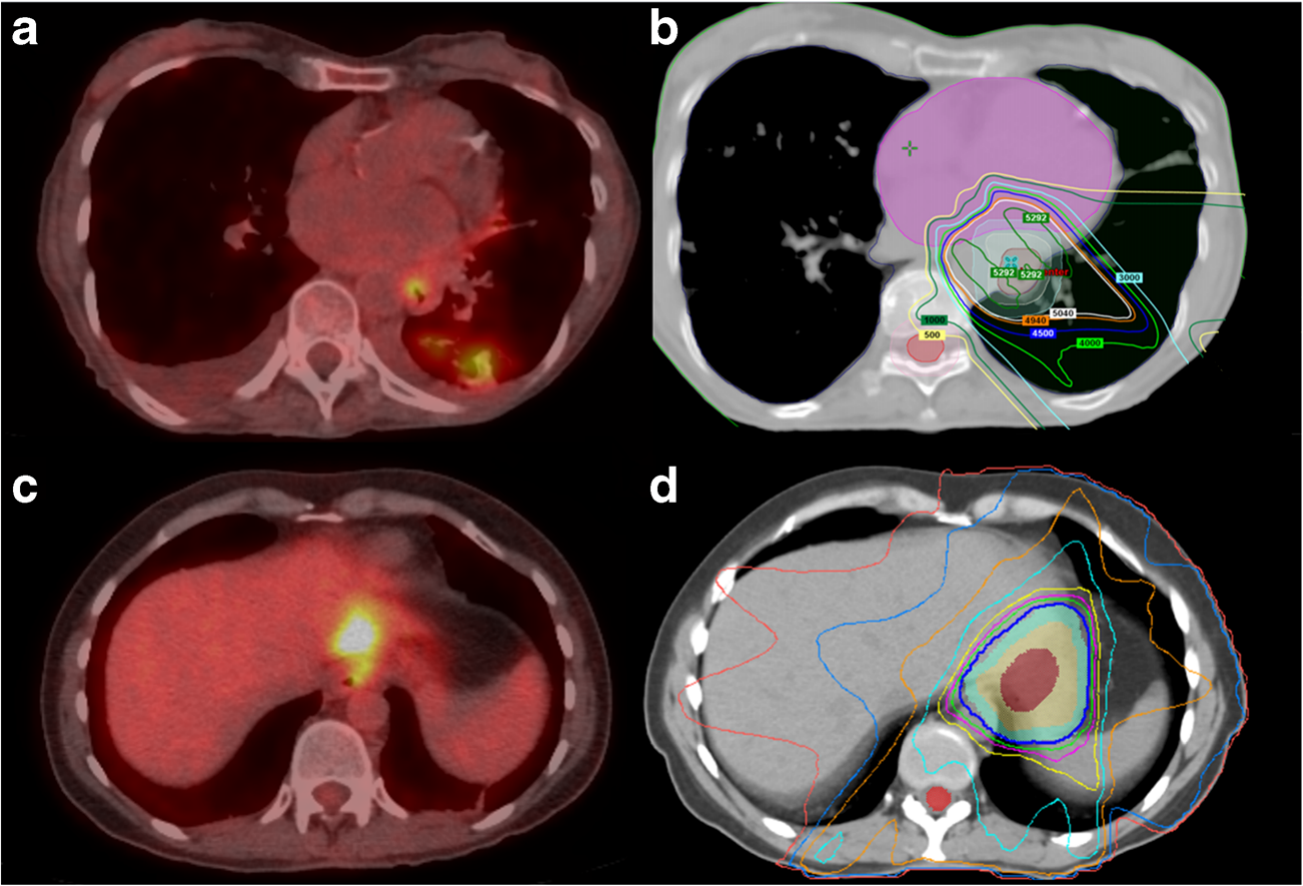
Examples of new non-malignant ^18^F-FDG avid lesions detected by ^18^F-FDG PET/CT restaging. (a): 78-year-old woman with squamous cell carcinoma of the esophagus treated with chemoradiation. The PET/CT image shows new opacities within the left lower lobe with corresponding areas of ^18^F-FDG activity. The new lesion was within the presumed radiation field (b) and the appearance was most compatible with radiation-induced pneumonitis (scan was regarded as ‘true negative’ for new metastatic disease). (c): 42-year-old female with adenocarcinoma of the distal esophagus who had undergone chemoradiotherapy. The PET/CT images show linear ^18^F-FDG accumulation within the lateral aspect of the left hepatic lobe. The new lesion was within the presumed radiation field (d) and was thought to be related to radiation therapy changes, which was confirmed with an MRI scan (scan was regarded as ‘false positive’ as additional imaging was required to exclude metastatic disease)

In 109 of 783 (13.9%) patients, new potential metastatic lesions were detected during PET/CT restaging. Of these patients, 65 (TP: 65/783; 8.3%) were confirmed to have true metastatic disease by histology (*n* = 21) or clinical follow-up (*n* = 44), and 44 were deemed to have false positive results (FP: 44/783; 5.6%) (see Figs. 2 and 3 for examples). The location and treatment of new metastatic lesions are presented in Table 2, and the location of false-positives in Fig. 1. Median overall survival of patients with interval metastasis was 6 months (95% CI: 4–8 months), compared to 59 months for patients without metastatic disease at restaging (47–70 months).

**Table 2:** Location and treatment of interval metastasis, on ^18^F-FDG PET/CT after neoadjuvant chemoradiotherapy

	n (%)
Location of interval metastasis	
Lung	18 (22)
Liver	17 (21)
Retroperitoneal	16 (20)
Bone	16 (20)
Supraclavicular LN	7 (8)
Other	7 (8)
Number of locations with recurrence	
1	49 (75)
>1	16 (25)
Type of management	
*Treatment focused on tumor reduction*	*40 (62)*
Chemotherapy	31 (78)
Radiotherapy	5 (12)
Chemoradiation	4 (10)
*Best supportive care*	*25 (38)*

In patients with no evidence of interval metastasis after initial restaging with ^18^F-FDG PET/CT, new metastatic lesions were identified in 22 (FN: 22/783; 2.8%) patients within 3 months of follow-up (Fig. 1). The resulting overall per-patient sensitivity and specificity of ^18^F-FDG PET/CT to detect interval metastasis was 74.7% (95% CI: 64.3–83.4%) and 93.7% (95% CI: 91.6–95.4%), respectively. Positive and negative predictive values of PET/CT were 59.6% (95% CI: 52.0–66.9%) and 96.8% (95% CI: 95.4–97.7%), respectively (Table 3).

**Table 3:** Diagnostic parameters of ^18^F-FDG PET/CT for the detection of interval metastasis

Parameter	^18^F-FDG PET/CT
Sensitivity (%) [95%CI]	65/87 (74.7%) [64.3–83.4]
Specificity (%) [95%CI]	652/696 (93.7%) [91.6–95.4]
Positive predictive value (%) [95%CI]	65/109 (59.6%) [52.0–66.9]
Negative predictive value (%) [95%CI]	652/674 (96.7%) [95.4–97.7]
Diagnostic accuracy	91.6%

### Pre-treatment prediction of interval metastasis

The univariable associations of clinical factors with interval metastasis after chemoradiotherapy are summarized in Table 1. After multivariable analysis, clinical nodal involvement (odds ratio [OR]: 2.91, 95% CI: 1.34–6.32), EUS-based tumor length ≥ 4 cm (OR: 2.68, 95% CI: 1.11–6.52), squamous cell tumor histology (OR: 1.65, 95% CI: 0.86–3.17) and baseline SUV_max_ ≥ 9.6 (OR: 1.66, 95% CI: 0.94–2.93) remained independently predictive for the occurrence of interval metastasis (Table 3). The discriminative ability of the final model was reasonable with an apparent C-statistic of 0.69, and 0.67 after adjustment for optimism. The shrinkage factor for the coefficients was 0.88. The adjusted β-coefficients of the prediction model for interval metastasis after shrinkage are presented in Table 3.

**Fig. 4:**
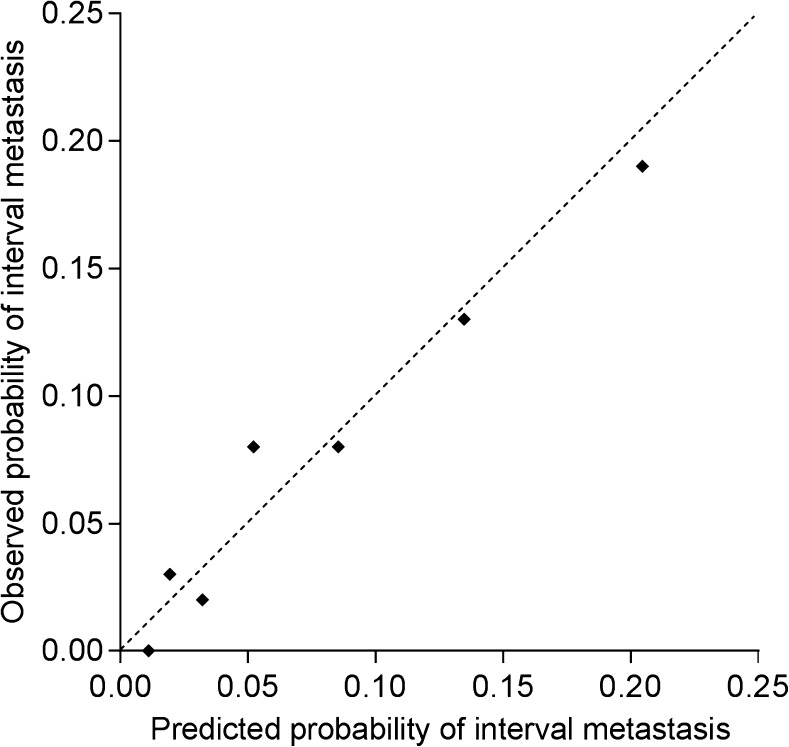
Calibration curve for predicted probability of interval metastasis for each unit of the risk score versus the observed frequency of interval metastasis

**Fig. 5:**
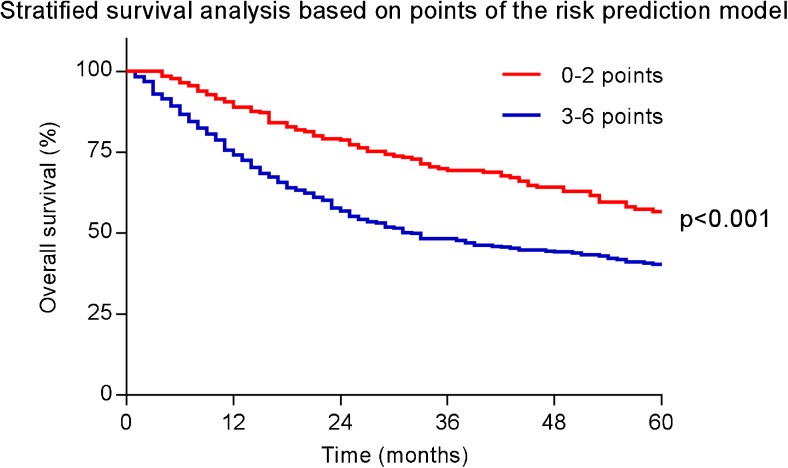
Risk prediction model for interval metastasis predicts overall survival in patients without interval metastasis after ^18^F-FDG PET/CT restaging

A practical prediction tool for the development of interval metastasis was developed based on the 4 predictors that remained in the final model. Based on the adjusted β-coefficients, each variable was converted into a corresponding number of points (multiplied by 2) rounded to its nearest integer. The total risk score was calculated by adding up the number of points obtained for each clinical predictor (cN status + EUS-based tumor length + tumor histology + baseline SUV_max_ = risk score; Table 4). In Table 4, also, the corresponding observed risk for interval metastasis can be found for the different risk scores. The correspondence between the predicted risk of interval metastasis by the risk score and actual observed interval metastasis indicated good calibration (Fig. 4). In patients without interval metastasis after ^18^F-FDG PET/CT restaging, the risk score was also significantly associated with survival (Fig. 5, *p* = 0.001).

**Table 4:** Risk prediction model for distant interval metastases

Characteristic	Odds-ratio (95% confidence interval)		Original regression coefficients		Adjusted regression coefficients		*p-value*	*points*
Clinical nodal stage (N+ vs. N0)	2.91 (1.34–6.32)		1.069		0.940		0.007	2
EUS-based tumor length (≥4.0 cm vs. <4.0 cm)	2.68 (1.11–6.52)		0.988		0.869		0.029	2
Tumor histology (squamous cell vs. adenocarcinoma)	1.65 (0.86–3.17)		0.501		0.440		0.132	1
SUV_max_ primary tumor at baseline (≥9.6 vs. <9.6)	1.66 (0.94–2.93)		0.509		0.448		0.078	1
Total number of points:	0	1	2	3	4	5	6	
Number of patients at risk:	81	31	140	94	165	225	47	
Risk of interval metastases (%):	1.1%	1.9%	3.2%	5.2%	8.5%	13.5%	20.5%	

Intercept: −4.425, shrinkage factor: 0.88

## Discussion

Findings in the current study demonstrate that ^18^F-FDG PET/CT restaging after neoadjuvant chemoradiotherapy detects interval metastases in 8% of oesophageal cancer patients, with a patient-based sensitivity and specificity of 75% and 94%, respectively. Independent risk factors for the development of interval metastases include clinical nodal involvement, EUS-based tumor length of ≥4 cm, squamous cell tumor histology, and baseline SUV_max_ of ≥9.6. Based on these findings, a prediction score was developed which may provide physicians a tool for objectively assessing the risk of interval metastasis in patients with oesophageal cancer.

Accurate preoperative detection of (interval) metastasis of oesophageal cancer is crucial for optimal selection of patients suitable for surgery. In patients with accurately detected interval metastasis, surgery is expected to provide no benefit in terms of survival, but rather to decrease quality of life due to highly morbid surgery with subsequent recovery time [28]. In this regard, the findings of the current study indicate that 8% of patients were spared a futile oesophagectomy as a result of our routine ^18^F-FDG PET/CT restaging protocol. Although previous studies on this topic have reported detection rates of interval metastasis ranging between 2% and 26%, guideline recommendations on restaging patients after neoadjuvant chemoradiotherapy for oesophageal cancer remain contradictory [10–12].

The incidence of interval metastasis in the current study is consistent with the results of previous reports [6–8]; however, there are some important other aspects of ^18^F-FDG PET/CT restaging that should be considered. Our findings indicate that in 86% of patients, no new lesion will be detected during restaging after chemoradiotherapy, indicating that limited impact on patient management is anticipated in the majority of patients. In another 6% of the patients, restaging results in false positive findings introducing unnecessary imaging and biopsy procedures. It should be noted that this work-up is associated with additional costs and that biopsy procedures are not without risks [29].

Consequently, a more individualized application of ^18^F-FDG PET/CT restaging could reduce the number of unbeneficial diagnostic tests. Yet, little is known about what patients are at risk for developing interval metastases, and the small number of patients in the previous mentioned studies precludes assessment of predictors for interval metastasis after neoadjuvant therapy [6–8, 15]. These findings encouraged us to develop a risk prediction score for interval metastases that may guide a more targeted application of ^18^F-FDG PET/CT restaging. This may especially be of interest for hospitals/regions with limited resources that have not yet implemented ^18^F-FDG PET/CT restaging in their routine clinical practice due to associated costs.

The proposed risk score - based on well-recognized prognostic factors [3, 20, 21, 24, 25, 30] - has reasonable predictive value and may guide clinical decision making. The data indicate that patients with low scores have limited risk of interval metastases, and that in these patients a restaging ^18^F-FDG PET/CT may be safely omitted without subjecting the patient to the risks of further diagnostic tests. Increasing the threshold for restaging patients with ^18^F-FDG PET/CT based on the proposed risk score will result in a further reduction of unnecessary additional scans and biopsies (false positives), but at the potential cost of missing interval metastasis (false negatives). Determining an appropriate threshold at which to initiate restaging will depend on patients’ and physicians’ judgments about the harm of missed interval metastasis versus unnecessary diagnostic tests and available resources.

The relatively high incidence (11%) of early disease progression already within 6 months after restaging (Fig. 1) suggests that small distant metastases, which are not detected by ^18^F-FDG PET/CT, may already have occurred at the time of restaging [31]. This indicates that while ^18^F-FDG PET/CT detects a substantial proportion of interval metastasis, it is sometimes insufficient to detect all early disease progression. Therefore, one may consider close monitoring of high-risk patients with additional (perioperative) restaging. This suggestion is supported by our finding that the risk score was also predictive for survival after initial restaging.

In the context of clinical decision-making with regard to those patients who are most likely to benefit from an oesophageal resection after chemoradiotherapy, the prediction of pathological response to neoadjuvant therapy may be another motivation to perform ^18^F-FDG PET/CT restaging. It has been suggested that preoperative identification of patients with a pathologic complete response – aided by information derived from ^18^F-FDG PET/CT - could enable a wait-and-see approach with omission of surgery [19, 32]. However, currently uncertainty continues to exist over the clinical benefit of ^18^F-FDG PET/CT with regard to the accuracy for differentiating between residual tumor and therapy induced inflammation after chemoradiotherapy [33–35]. Other reasons to perform a restaging scan include surgical planning (notable for GEJ tumors).

As discussed, the false positive rate of 6% during ^18^F-FDG PET/CT restaging was substantial, with the lungs and liver as the most frequent affected sites. This confirms previous findings in literature, with reported false positive rates ranging between 0% and 10% [6, 36] and liver and lung as the most commonly affected sites [29, 37]. This is likely caused by radiation-induced disease that may falsely indicate disease progression (Figs. 2 and 3). Previous studies evaluating new FDG-avid hepatic lesions within the presumed radiation field of patients with oesophageal cancer demonstrated that these lesions generally reflect radiation-induced liver disease rather than metastatic disease [37–39]. Evaluation of radiation fields may, therefore, aid in the assessment of restaging ^18^F-FDG PET/CT scans and further clinical decision-making [37].

Potential limitations of this study are that it used follow-up information as a reference standard, which is challenging because follow-up should be long enough to allow hidden cases of disease to progress to a detectable stage, while it should be short enough to prevent new cases that develop after restaging to be detected. Because the length of follow-up to determine disease-status is arbitrary, reported diagnostic accuracy measures may vary in cases of different follow-up lengths. Second, histological biopsy was not performed in all patients with suspected interval metastasis, which may have introduced reference test bias. Third, quantitative imaging values such as SUV may be biased by many factors related to clinical protocols and PET system settings, many of which are center or manufacturer dependent. Therefore, future studies that use quantitative imaging for prognostic modeling are encouraged to control biases through standardization of imaging procedures by using harmonization programs (e.g. Quantitative Imaging Biomarkers Alliance [40]). Furthermore, the current study represents a single-institution analysis where findings in general may not be generalizable to other centers. Therefore, external validation of the developed risk prediction score is recommended to determine generalizability [41].

Despite the aforementioned limitations, major strengths of this study include that it is the largest study so far to assess the diagnostic performance of ^18^F-FDG PET/CT for the detection of interval metastases. Furthermore, it provides the first clinically applicable risk prediction score for interval metastasis after chemoradiotherapy for oesophageal cancer.

## Conclusion

^18^F-FDG PET/CT restaging detects true distant interval metastases in 8.3% of patients after chemoradiotherapy for oesophageal cancer. The provided prediction score stratifies risk of developing interval metastasis, and could be used to prioritize additional restaging modalities for patients most likely to benefit. Centers that do not routinely perform ^18^F-FDG PET/CT restaging, should at least consider scans for patients at high risk of interval metastases.
